# An aging-related immune landscape in the hematopoietic immune system

**DOI:** 10.1186/s12979-023-00403-2

**Published:** 2024-01-02

**Authors:** Jianjie Lv, Chun Zhang, Xiuxing Liu, Chenyang Gu, Yidan Liu, Yuehan Gao, Zhaohao Huang, Qi Jiang, Binyao Chen, Daquan He, Tianfu Wang, Zhuping Xu, Wenru Su

**Affiliations:** 1 State Key Laboratory of Ophthalmology, Guangdong Provincial Key Laboratory of Ophthalmology and Visual Science, Zhongshan Ophthalmic Center, Sun Yat-Sen University, Guangzhou, 510060 China; 2 Department of Ophthalmology, West China Hospital, Sichuan University, Chengdu, 610041 Sichuan China

**Keywords:** Hematopoietic immune system, Neutrophils, Aging, Spleen, Bone marrow, Myeloid cells, Hematopoietic stem cells, Single-cell sequencing, Single-cell flow cytometry

## Abstract

**Background:**

Aging is a holistic change that has a major impact on the immune system, and immunosenescence contributes to the overall progression of aging. The bone marrow is the most important hematopoietic immune organ, while the spleen, as the most important extramedullary hematopoietic immune organ, maintains homeostasis of the human hematopoietic immune system (HIS) in cooperation with the bone marrow. However, the overall changes in the HIS during aging have not been described. Here, we describe a hematopoietic immune map of the spleen and bone marrow of young and old mice using single-cell sequencing and flow cytometry techniques.

**Results:**

We observed extensive, complex changes in the HIS during aging. Compared with young mice, the immune cells of aged mice showed a marked tendency toward myeloid differentiation, with the neutrophil population accounting for a significant proportion of this response. In this change, hypoxia-inducible factor 1-alpha (Hif1α) was significantly overexpressed, and this enhanced the immune efficacy and inflammatory response of neutrophils. Our research revealed that during the aging process, hematopoietic stem cells undergo significant changes in function and composition, and their polymorphism and differentiation abilities are downregulated. Moreover, we found that the highly responsive CD62L + HSCs were obviously downregulated in aging, suggesting that they may play an important role in the aging process.

**Conclusions:**

Overall, aging extensively alters the cellular composition and function of the HIS. These findings could potentially give high-dimensional insights and enable more accurate functional and developmental analyses as well as immune monitoring in HIS aging.

**Supplementary Information:**

The online version contains supplementary material available at 10.1186/s12979-023-00403-2.

## Background

Aging is a universal, progressive, and irreversible biological process that occurs simultaneously in the cells, tissues, and organs. Although life expectancy has increased over the past 200 years owing to medical advances, the medical and social problems associated with aging have become increasingly serious [1]. As aging progresses, the normal physiological functions of several systems, including the immune system, gradually become dysfunctional [2, 3]. The accumulation of senescent cells and their senescence-associated secretory phenotype (SASP) have been linked to a decline in physical function and structural changes [4]. Aging increases the risk of various diseases, including cancer, diabetes, and cardiovascular, neurodegenerative, and autoimmune diseases and is ultimately associated with an increased risk of death [5–8]. Overall, aging broadly affects normal physiological functions and is associated with susceptibility to heterogeneous diseases.

The immune system, that resists invasion by external pathogens and maintains normal physiological functions, is strongly affected by aging, as the composition and function of highly specific immune cell populations changes with age. Aging alters the relative abundances of B cells (BC), T cells (TC), natural killer (NK) cells, dendritic cells (DCs), macrophages, neutrophils, and other immune cell subsets [9]. Aging-induced immune system dysfunction is associated with immune senescence and inflammation [10]. We previously reported that aging promotes the polarization of human circulating immune cells and the increased expression of inflammatory genes as well as SARS-CoV-2 susceptibility genes, and this may explain the vulnerability of the elderly to COVID-19 [11]. In addition, aging alters the function of immune cells, as in stroke, where aging leads to neutrophil dysfunction and the rapid accumulation of pro-inflammatory neutrophils in the bloodstream and ischemic brain, exacerbating the disease [12]. More importantly, aging profoundly affects the hematopoietic and immune systems that are important for the regeneration and distribution of immune cells in peripheral blood and tissues. It manifests as aberrant hematopoietic stem cell (HSC) differentiation potential, leading to an age-dependent decline in hematopoiesis and immune function [13]. Therefore, it is necessary to systematically study the effects of aging on the hematopoietic and immune systems.

The bone marrow and spleen, the main organs of hematopoietic and immune system (HIS), contain a complete repertoire of immune cells at different developmental stages and are closely related to the immune response. These two organs contain HSCs and differentiated progenitor cells, as well as further differentiated immune cells, including NK cells, neutrophils, basophils, monocytes, DCs, B cells, and T cells [14, 15]. Recent in vivo studies have shown that aging reduces the lymphocyte differentiation potential of HSCs in the bone marrow as well as promoting age-related anemia and dysplasia [16]. Kira et al. conducted an analysis of hematopoietic stem cells in the bone marrow of mice at various stages of aging. Their study revealed that hematopoietic stem cells start to exhibit aging traits during middle age (9–12 months). Furthermore, the researchers identified the decline of the longevity-related molecule IGF1 as a contributing factor to the aging of hematopoietic stem cells [17]. And in vitro studies have also shown that aging contributes to the loss of HSC protein polarity and lymphoid/myeloid differentiation skewing [18]. However, these studies have mainly focused on the effects of aging on bone marrow HSCs or HSCs in a cell culture environment in vitro. The spleen is an important hematopoietic and immune organ that performs hematopoietic functions in several pathological conditions. In addition, spleen is also the important organ and site that facilitates cellular and humoral immune responses [19]. DenisA et al. employed single-cell techniques to investigate the alterations in immune cells in the spleen and other organs of aging mice. They identified an age-related subgroup of GZMK + CD8 + T cells, but did not provide comprehensive descriptions of other cell types, particularly HSCs [20]. Therefore, a study that comprehensively characterizes changes in HIS cells during aging is highly desirable.

With high accuracy and specificity, the high-throughput single-cell RNA sequencing (scRNA-seq) technology has advantages in uncovering changes in the transcriptome at the level of single cells, thus allowing us to better explore the biological processes associated with aging [9, 21]. Our previous study employed single-cell technologies on human peripheral blood mononuclear cells and murine lymph nodes and revealed the effects of aging on the immune system [22]. Therefore, we used scRNA-seq to comprehensively characterize the properties of HIS cells in young (2–3 weeks old) and old (20–22 months old) mice. Using flow cytometry and scRNA-seq methodology, we found that aging alters the composition of cellular subsets, gene expression characteristics, enrichment pathways, transcriptional regulatory networks, and intercellular interactions. Aging impairs the stemness and cellular activity of HSC and disrupts their differentiation balance. Aging maintains the body in a proinflammatory state and strongly affects neutrophils. These findings provide a comprehensive landscape of the effects of aging on the hematopoietic immune system (HIS) and expand our understanding of aging as a risk factor for inflammation, autoimmune diseases, and age-related disorders.

## Results

### Overall effects of aging on the characterization of the hematopoietic immune system (HIS)

**Fig. 1:**
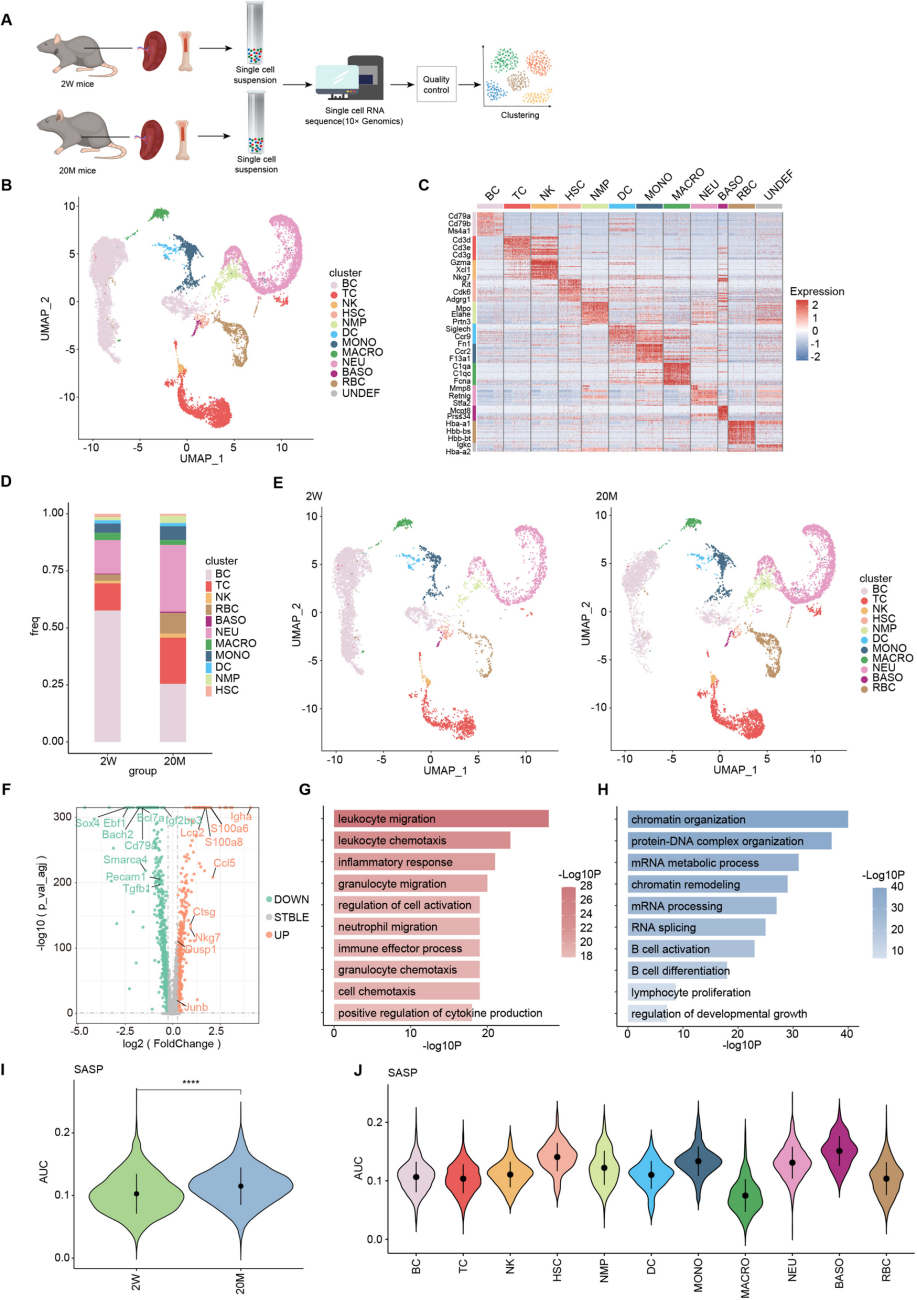
Overall effects of aging on the characterization of Hematopoietic Immune system. **A** Schematic diagram of experimental design for scRNA-seq analysis of bone marrow and spleen cells from 2W and 20M mice group. **B** UMAP plot showing the immune cell types of hematopoietic Immune system in scRNA-seq. **C** A heatmap showing scaled expression of discriminative gene sets for each cell type. **D** Bar chart showing the proportion of immune cell types respectively between the two groups of mice. **E** UMAP plot showing the immune cell types of the hematopoietic immune system in scRNA-seq respectively between the two groups of mice. **F** Volcano plot showing the up and downregulated DEGs between the two groups of mice. **G** Representative GO biological processes and pathways enriched in upregulated DEGs in 20M mice. **H** Representative GO biological processes and pathways enriched in downregulated DEGs in 20M mice. **I** Violin plot showing the SASP signaling score between the two groups of mice. **J** Violin plot showing the SASP signaling score among cell types in 20M mice. Significance in I was calculated using the Wilcoxon test; ****P < 0.0001

Total bone marrow was obtained from the femur, tibia, and whole spleen of young and old mice. After digestion and lysis of the erythrocytes, bone marrow and spleen cell suspensions were collected for scRNA-seq (Fig. 1A). After clustering based on previously reported cell markers, all cells were classified into BC, TC, NK, Hematopoietic stem cells (HSC), neutrophil-myeloid progenitor (NMP), DC, monocytes (MONO), macrophages (MAC), neutrophils (NEU), basophils (BASO), red blood cells (RBC), and undefined cells (UNDEF) (Fig. 1B, C and S1A). Compared to young mice, the proportion of cell subgroups in the HIS (comprised of the bone marrow and spleen) of aging mice changed dramatically. With aging, the proportion of lymphocytes decreased markedly, including a pronounced decrease in the proportion of B cells and an increase in the proportion of T and NK cells, whereas the proportion of myeloid cells increased obviously, with an increase in the proportion of BASO, MONO, and NEU, and a decrease in the proportion of MAC (Fig. 1D, E). To further explore the effects of aging, we performed DEG analysis on these cell populations between the 2W and 20M mouse groups and found that cells from old mice expressed higher levels of inflammation-related genes (including the S100 family, AP-1 family, Igha, Ctsg, Ccl5, and Nkg7) and lower levels of growth- and development-related genes (including Sox4, Tgfb1, and Ebf1) than those from young mice (Fig. 1F). In addition, counting the number of upregulated and downregulated DEGs in the cell subgroups revealed a higher number of DEGs in BC, TC, NEU, and MONO, suggesting that aging may have a greater impact on these cell subgroups (Fig. S1B).

To better demonstrate the effects of aging on cell function, we conducted enrichment analyses of the upregulated and downregulated DEGs to investigate their biological functions. The results indicated that the upregulated DEGs were mainly enriched in signaling pathways associated with the inflammatory response (such as “inflammatory response,” “immune effector process,” and “positive regulation of cytokine production”) and the immune cell migration and chemotaxis processes (such as “leukocyte migration,” “leukocyte chemotaxis,” and “granulocyte migration”). The downregulated DEGs were mainly enriched in signaling pathways associated with cell growth (such as “chromatin organization,” “protein-DNA complex organization,” and “mRNA metabolic process”) and the cell activation and differentiation processes (such as “B cell activation,” “B cell differentiation,” and “lymphocyte proliferation”) (Fig. 1G, H). The senescence-associated secretory phenotype (SASP) displayed by cells impacts the alterations seen with aging [23]. Therefore, we compared the SASP scores of young and old mice and found that old mice had higher SASP scores than young mice (Fig. 1I). SASP scoring of each cell subgroup in old mice showed that HSC and myeloid cells (including MONO, NEU, and BASO) had higher SASP scores (Fig. 1J). The SASP affects the expression of cytokines and chemokines involved in the inflammatory response. Therefore, we further evaluated and compared the inflammatory response pathway scores between the groups and cell subgroups. We found that the inflammatory response pathway scores of old mice were higher than those of young mice, in which the scores of NEU, MONO, and BASO were higher than those of other cells in old mice, indicating that aging causes the cells in the HIS, especially myeloid cells, to be more involved in inflammatory response pathways, and this may further contribute to aging progression (Fig. S1C, D). Overall, we demonstrated the effects of aging on cell proportions and functions in the HIS and found that aging promoted inflammatory responses and inhibited cell growth and developmental processes.

### Aging alters the composition and function of T cells in the HIS

**Fig. 2:**
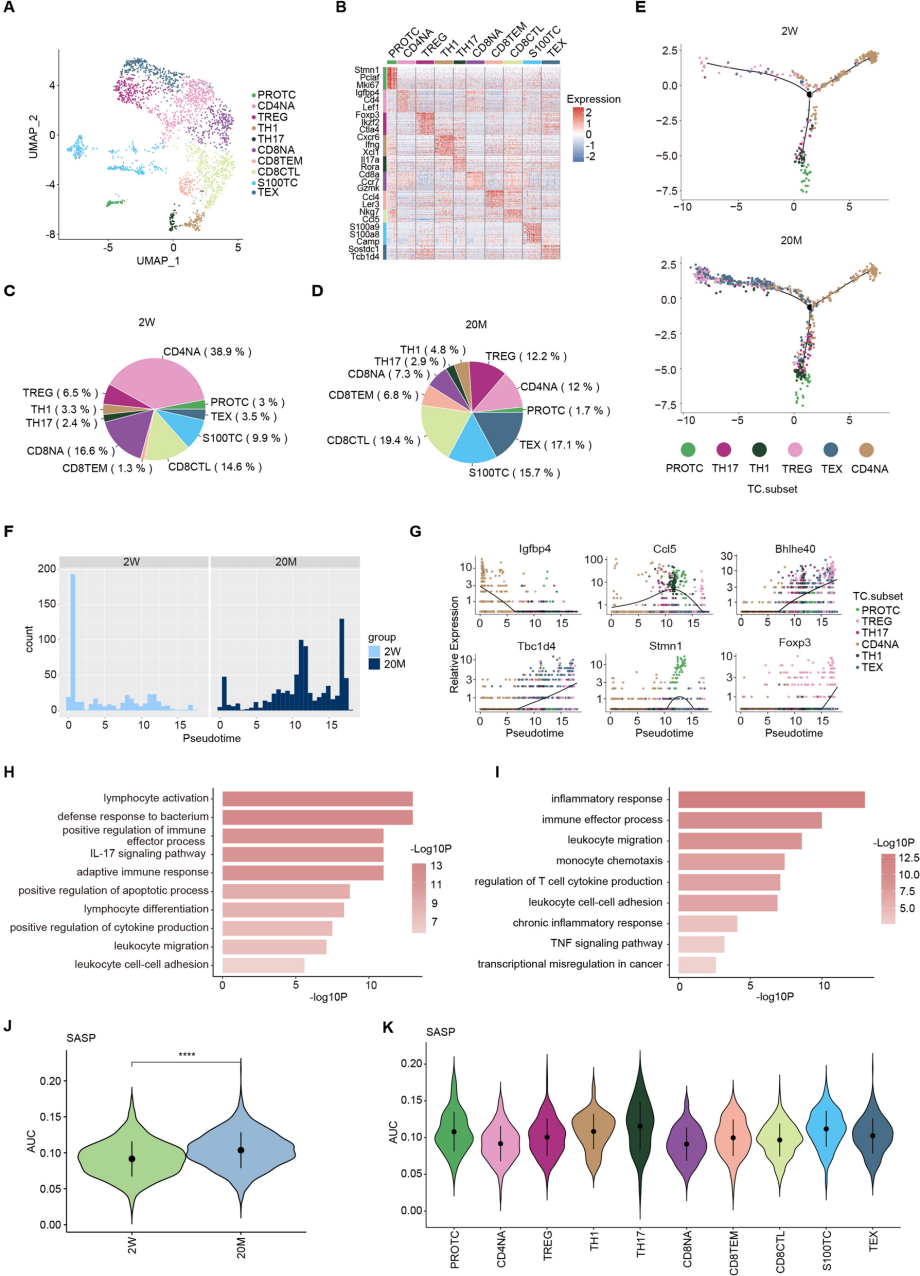
Aging alters the composition and function of T cells in the HIS. **A** UMAP plot showing the TC subsets of the hematopoietic immune system in scRNA-seq. **B** A heatmap showing scaled expression of discriminative gene sets for TC subsets. **C** Pie charts showing the proportion of TC subsets in 2W mice. **D** Pie charts showing the proportion of TC subsets in 20M mice. **E** Pseudotime trajectory analysis of CD4 + T cells subsets between two group. Cells are arranged by Pseudotime. **F** Percentages of CD4 + T cells along the pseudotime for two groups. **G** Expression transition of Igfbp4, Ccl5, Bhlhe40, Tbc1d4, Stmn1, Foxp3 in CD4 + T cells subsets along the pseudotime. **H** Representative GO biological process and pathways enriched in upregulated DEGs in CD4 + T cells. **I** Representative GO biological process and pathways enriched in upregulated DEGs in CD8 + T cells. **J** Violin plot showing the SASP signaling score between two groups in TC. **K** Violin plot showing the SASP signaling score among cell types in TC of 20M group. Significance in J was calculated using wilcoxon test; ****P < 0.0001

To further explore the changes in immune cells during aging, we analyzed cell subgroups. All T cells were clustered and classified into proliferative T cells (PROTC), naïve CD4 + T cells (CD4NA), regulatory T cells (TREG), T helper 1 cells (TH1), T helper 17 cells (TH17), naïve CD8 + T cells (CD8NA), CD8 + effector memory T cells (CD8TEM), CD8 + T cells with cytotoxic activity (CD8CTL), S100 T cells (S100TC), exhausted T cells (TEX), according to cellular markers (Fig. 2A, B). The changes in the proportion of T cells showed a trend consistent with aging. Compared with young mice, old mice had markedly lower proportions of CD4NA, CD8NA, and PROTC, and higher proportions of TEX, TREG, and S100TC. Naïve T cells dominated in young mice, whereas effector and exhausted T cells dominated in old mice (Fig. 2C, D, and S2A). To explore the reasons for this difference in terms of cell development and differentiation processes, we performed a pseudotime analysis in CD4 + T cells and CD8 + T cells. We observed a trajectory from naïve CD4 + T cells to exhausted T cells, and naïve CD8 + T cells to CD8CTL, respectively. Compared with young mice, old mice had more T cells at the end of the trajectory. Specifically, older mice had less CD4NA at the beginning of the trajectory but more TREG and TEX at the end of the trajectory, further indicating that aging maintained more T cells in a highly differentiated state (Fig. 2E, F, and S2B). Six genes with obvious differences in T cells were selected and visualized based on their pseudotimes. Over time, the expression of naïve-related Igfbp4 gradually decreased, whereas the expression of effector and exhaust-related Foxp3 and Tbc1d4 gradually increased. Moreover, the expression of inflammation-related genes Ccl5 and Bhlhe40 increased gradually with time. Among these, Igfbp4 was highly expressed in the young group, whereas other genes were highly expressed in the old group (Fig. 2G and S2C). Enrichment analyses showed that lymphocyte activation and differentiation, adaptive immune response, and inflammatory response processes were upregulated, whereas cell proliferation and differentiation processes were downregulated in the CD4 + T cells of old mice (Fig. 2H and S2D). These findings imply that aging mice are in a state of chronic inflammation involving CD4 + T cells.

Pseudotime analysis of CD8 + T cells also showed similar results to CD4 + T cells, with a decreased proportion of naïve CD8 + T cells and an increased proportion of terminal effector CD8 + T cells in old mice (Fig. S2E-G). Continued enrichment analysis of DEGs from CD8 + T cells in old mice showed that these upregulated DEGs were predominantly enriched in pathways associated with inflammatory responses and immune effector processes, whereas the downregulated DEGs were predominantly enriched in biological processes associated with cell proliferation and differentiation (Fig. 2I and S2H). Finally, we used inter-subset SASP scores to investigate the differences in the effects of aging on T cell subsets and found that old mice had higher scores, and PROTC, TH17, and S100TC had the greatest contribution, further demonstrating that aging puts T cells in a highly differentiated state and they become more involved in inflammatory response processes (Fig. 2J-K). In summary, aging increases the number of T cells in a highly differentiated state that are more involved in immune and inflammatory responses against external pathogens.

### Aging enhances the immune response of B cells, but reduces the response to new antigens

**Fig. 3:**
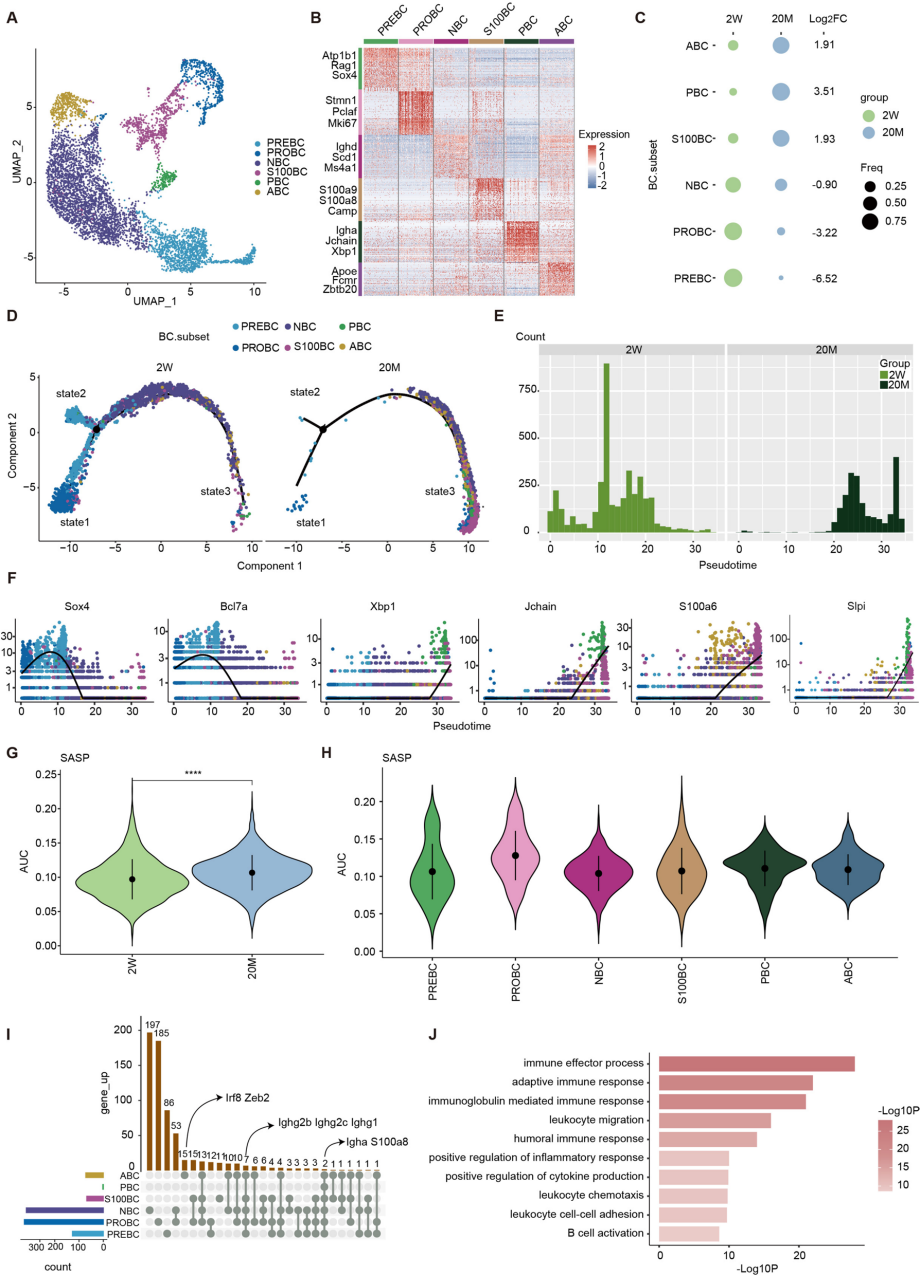
Aging enhances the immune response of B cells, but reduces the response to new antigens. **A** UMAP plot showing the BC subsets of hematopoietic Immune system in scRNA-seq. **B** The heatmap showing scaled expression of discriminative gene sets for BC subsets. **C** The dot plot showing the proportion of BC subsets between two groups. **D** Pseudotime trajectory analysis of BC subsets between two groups. Cells are arranged by Pseudotime. **E** Percentages of BC along the pseudotime for two groups. **F** Expression transition of Sox4, Bcl7a, Xbp1, Jchain, S100a6, and Slpi in BC subsets along the pseudotime. **G** Violin plot showing the SASP signaling score between two groups in BC. **H** Violin plot showing the SASP signaling score among cell types in TC of 20M group. **I** UpSet plot showing the integrated comparative analysis of upregulated DEGs in BC subsets. **J** Representative GO biological process and pathways enriched in upregulated DEGs in BC. Significance in J was calculated using wilcoxon test; ****P < 0.0001

Next, we conducted a systematic analysis of B cells that were classified into six clusters according to specific cellular markers: precursor B cells (PREBC), progenitor B cells (PROBC), naïve B cells (NBC), S100 B cells (S100BC), plasma cells (PBC) and age-associated B cells (ABC) (Fig. 3A-B). A comparison of the proportions of B cell subsets in the two age groups indicated that the proportion of terminally differentiated B cells (ABC, PBC, and S100BC) was 2–3 times higher in old mice than in young mice, whereas the proportion of developing B cells (PREBC, PROBC, and NBC) was significantly reduced (Fig. 3C and S3A). As with the T cells, we performed a pseudotime analysis of these six B cell subsets to explore the effects of aging on their differentiation characteristics over time. Unlike young mice, in which B cells are mostly located at the beginning of the cell trajectory, B cells in old mice are mostly distributed at the end of the cell trajectory. Further visualization of the distribution of B cells with pseudotime showed that more S100BC, ABC, and PBC were present at the end of the cell trajectory in old mice (Fig. 3D-E and S3B). Counting the proportions of cells in each state revealed that old mice had a higher proportion of effector B cells and fewer precursor and naïve B cells at the terminal state-3 of the trajectory (Fig. S3C). Compared to T cells, in young mice, B cells had a more mature functional state, whereas in old mice, B cells contained fewer naïve B cells, indicating a weakened response to new foreign antigens (Fig. 3D, E). In addition, the expression levels of B cell development-related genes (Sox4 and Bcl7a) that were highly expressed in the young group, decreased over time, whereas the expression levels of genes involved in the immune response of B cells (Xbp1, Jchain, S100a6, and Slpi) that were highly expressed in the old mouse group, increased over time (Fig. 3F and S3D).

Moreover, the SASP scores of B cells in old mice were higher than those in young mice. Further SASP scoring of B cell subsets in old mice resulted in PROBC having higher SASP scores, indicating that it may be impacted more by aging (Fig. 3G-H). The inflammatory response pathway score for BC in old mice was higher than that in young mice (Fig. S3E). S100BC and ABC showed higher inflammatory response pathway scores in the old mice (Fig. S3F). Next, we sought to determine whether classical inflammatory and immune response-related genes were regulated by aging in a cell type-specific manner. Upset diagrams integrating the up-regulated DEGs in B cell subsets of old mice showed that the inflammatory response-related genes Igha and S100a8 were up-regulated in all B cells [24, 25]. The immune system signaling pathway-related genes Irf8 and Zeb2 [26] were upregulated in ABC (Fig. 3I). Functional enrichment analysis of these upregulated DEGs further supported our hypothesis that aging enables B cells to participate as effector cells in the immune response and drive inflammatory responses (Fig. 3J).

### The number and functional status of myeloid cells, especially neutrophils, markedly increases with aging

**Fig. 4:**
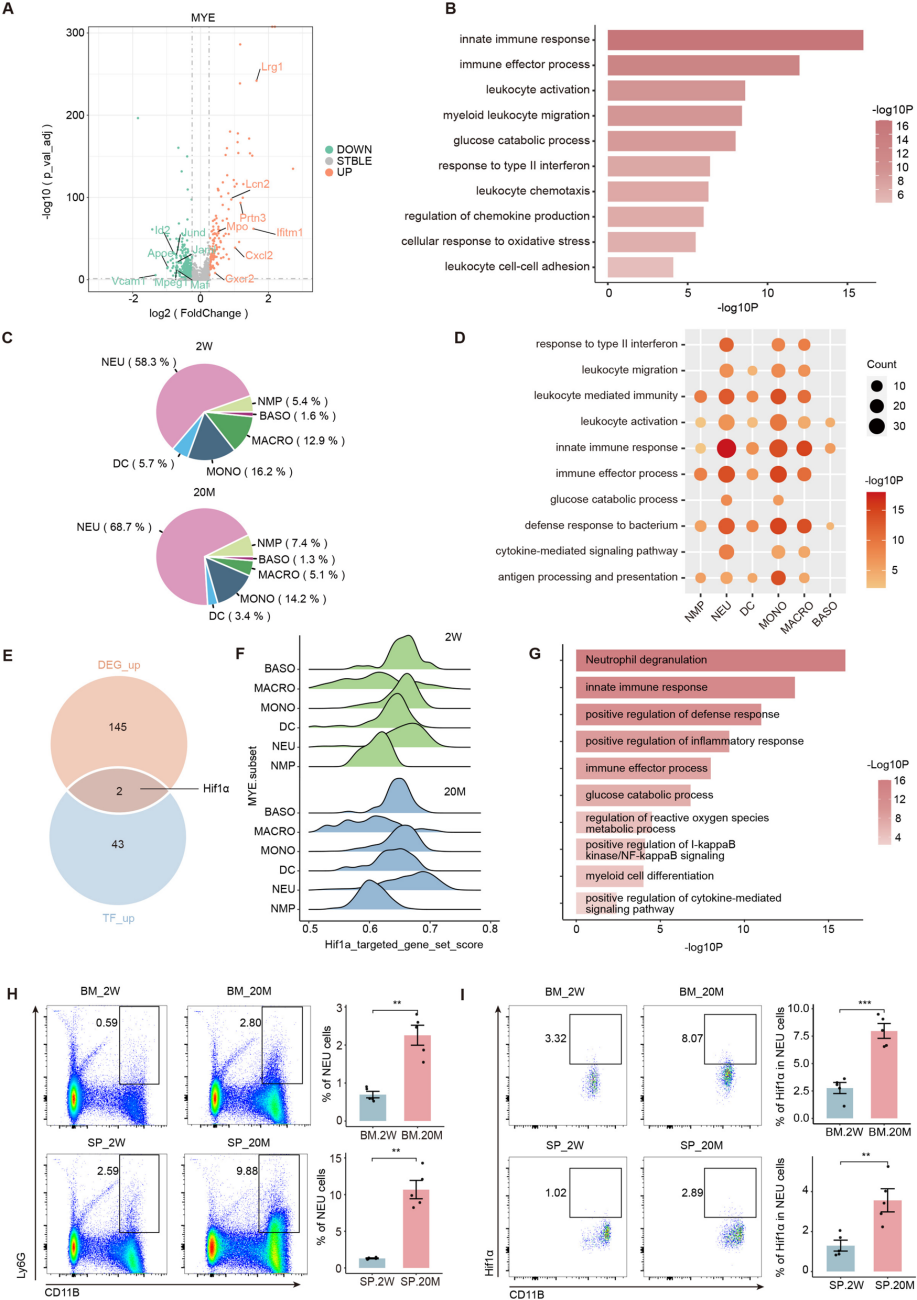
The number and functional status of myeloid cells, especially neutrophils, markedly increases with aging. **A** Volcano plot showing the up or downregulated DEGs in MYE. **B** Representative GO biological process and pathways enriched in upregulated DEGs in MYE. **C** The pie chart showing the proportion of MYE subsets between two groups. **D** Representative GO biological process and pathways enriched in upregulated DEGs among MYE subsets. **E** Venn diagram showing the interaction of upregulated DEGs and upregulated TFs in MYE. **F** Ridge plots showing the scores of the Hif1a_targeted_ gene_set among MYE subsets between two groups. **G** Representative GO biological process and pathways enriched in the intersect of Hif1a_targeted_ gene_set and upregulated DEGs in MYE cells. **H** The flow cytometry histograms (left) and column charts (right) showing the ration of NEU in bone marrow (up) and spleen (down) (*n* = 5/group). **I** The flow cytometry histograms (left) and column charts (right) showing the level of Hif1-α in NEU in bone marrow (up) and spleen (down) (*n* = 5/group). Significance in H-I was calculated using Student’s t test; *P < 0.05, ***P < 0.001, ****P < 0.0001

As identified in the SASP scoring results above, myeloid cells were more affected by aging (Fig. 1J). Genes such as Lrg1, Lcn2, and Prtn3 were upregulated in myeloid cells with aging, and myeloid cells were more involved in multiple immune effector processes as well as leukocyte migration and chemotaxis, whereas genes such as Id2, Jund, and Vcam1 were downregulated, decreasing their involvement in cell proliferation and differentiation (Fig. 4A, B, and S4A). Additionally, the effects of aging on the proportion of myeloid cells should not be ignored. In contrast to lymphocytes, old mice not only had an increased proportion of neutrophils but also an increased proportion of their precursor cells, NMP, indicating a tendency to differentiate into myeloid cells with aging (Fig. 4C and S4B). We further explored the biological functions of the bone marrow cell subsets. We visualized and analyzed subsets of cellular DEGs and found an upregulation of the S100 family and Lcn2 genes in mature myeloid cells. In addition, many genes related to the immune response and cytotoxic molecules (such as Ifitm3, Mpo, and Igkc) were upregulated in the most important NEU subgroup with aging (Fig. S4C). Functional enrichment analysis of myeloid cell subsets with upregulated DEGs showed that NEU and MONO exerted stronger biological functions, including immune responses and bactericidal processes, in old mice (Fig. 4D). Moreover, numerous genes related to mRNA processing and transcriptional activation (Ifg2bp3, Eif5, and Id2) were downregulated in NEU and MONO mice with aging (Fig. S4D). The downregulated DEGs in myeloid cells, especially NEU and MONO, were mostly enriched in cell proliferation- and differentiation-related pathways, such as regulation of the MAPK cascade, chromatin organization, and myeloid cell differentiation (Fig. S4E). These findings indicated that myeloid cells in old mice have a higher differentiation status and play a more robust role in immune effector processes and inflammatory responses. To explore the reasons for these changes, we performed transcription factor analysis on myeloid cells to further investigate the upstream mechanisms. We found that in the top 15 activated TFs, the transcription factors Zfp, Klf10, and Zbtb37 that are associated with RNA polymerase and DNA transcription were elevated in the young mice [27, 28] whereas the immune activation-associated transcription factors Cebpb and hypoxia-inducible factor 1-alpha (Hif1α) were significantly elevated in the old mice (Fig. S4F) [29, 30]. These upregulated TFs and up-regulated DEGs in all myeloid cells identified a potential target gene, Hif1α, that can regulate neutrophil survival through nuclear factor-kappa B (NF-κB) activation and reactive oxygen species (ROS) production (Fig. 4E-G). Also, Hif1α target-gene set scoring of myeloid cells from the two mouse groups further demonstrated a greater effect of aging on Hif1α and its target genes in NEUs (Fig. 4F). Additionally, functional enrichment analysis of up-regulated Hif1α target genes in NEU was conducted to show that the pathway was mainly enriched in neutrophil effector processes involving neutrophil degranulation, immune response, and inflammatory response (Fig. 4G). The inflammatory response process scores of myeloid cells in old mice were higher than those in young mice, with NEUs of old mice having the highest scores, reinforcing previous findings (Fig. S4G-H).

Consistent with our analysis above, the proportion of NEUs and their expression of Hif1α in the bone marrow and spleen of young and old mice, respectively, was verified by flow cytometry to reveal that there were more NEUs in the old mice and that they expressed higher levels of Hif1α (Fig. 4H-I, S7G). Overall, aging also has a great impact on the proportion and function of myeloid cells in the bone marrow and spleen, especially by increasing the proportion and number of NEU that are involved in immune effector processes and defense against pathogen invasion. Furthermore, we identified the potential target gene Hif1α that regulates neutrophil function after aging and this will help to deepen our understanding of the relationship between aging and neutrophils.

### Aging affects the differentiation potential and direction of differentiation of HSCs in the HIS

**Fig. 5:**
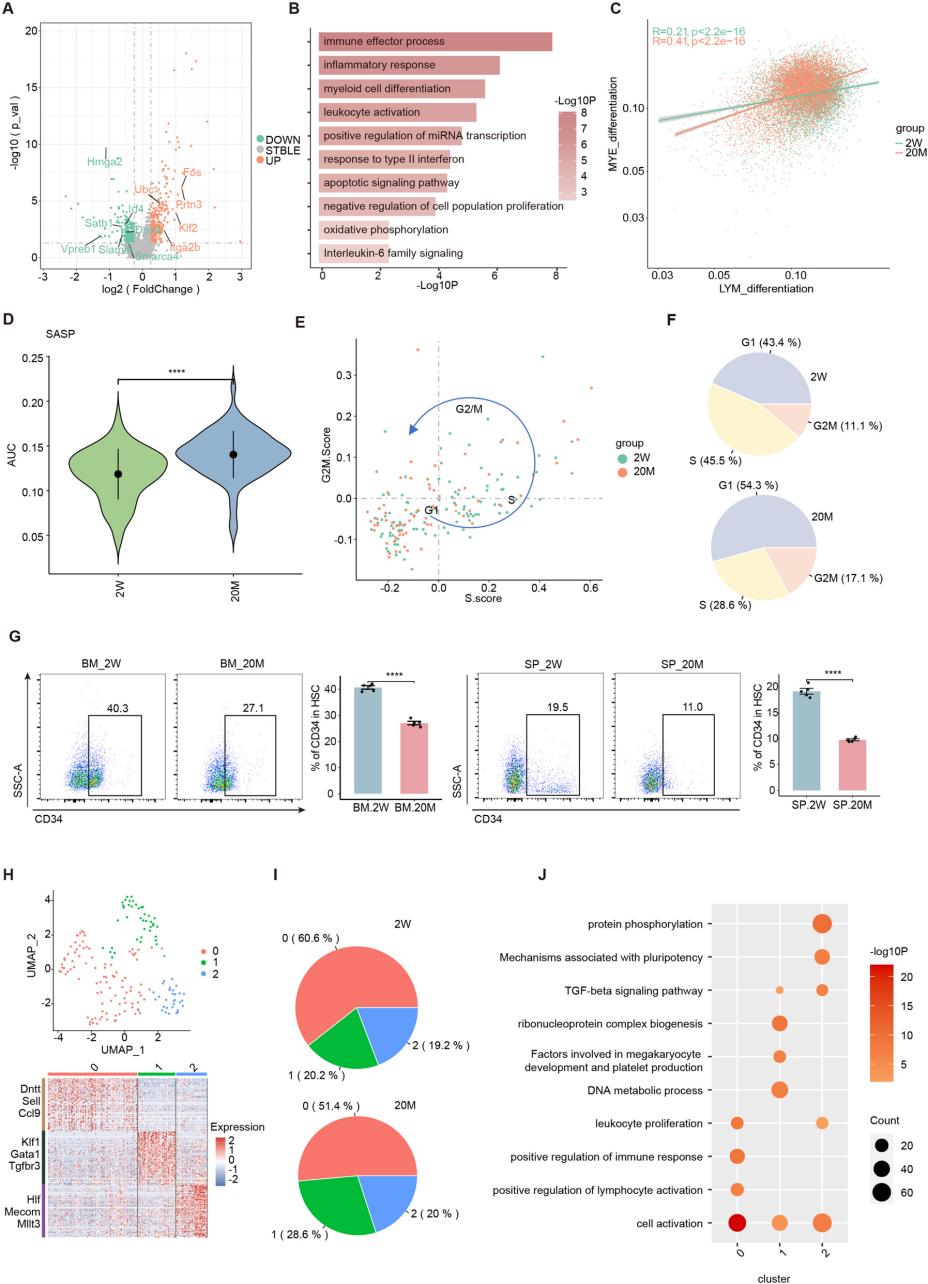
Aging affects the differentiation potential and direction of differentiation of HSCs in the HIS. **A** Volcano plot showing the up or downregulated DEGs in HSC. **B** Representative GO biological process and pathways enriched in upregulated DEGs in HSC. **C** The dot plot showing the lymphoid or myeloid differentiation scores of hematopoietic stem cells, lymphocytes, and myeloid cells. **D** Violin plot showing the SASP signaling score between two groups in HSC. **E** Classification of HSC from the three stages into the quiescent phase and other cycling phases (G1, S, and G2M) based on the cell-cycle score of G1/S and G2/M gene sets. **F** The pie chart showing the proportion of different cell_cycle statuses of HSC between two groups. **G** The flow cytometry histograms (left) and column charts (right) showing the percentage of LSK CD34 + HSC cells in bone marrow and spleen (*n* = 5/group). **H** UMAP plot showing the HSC clusters of hematopoietic Immune system in scRNA-seq (up). The heatmap showing scaled expression of discriminative gene sets for HSC clusters. **I** The pie chart showing the proportion of HSC clusters between two groups. J. Representative GO biological process and pathways enriched in the discriminative gene sets among HSC clusters. Significance in D was calculated using wilcoxon test; ****P < 0.0001. Significance in G was calculated using Student’s t test; **P < 0.01, ***P < 0.001, ****P < 0.0001

As major immune and hematopoietic organs, the bone marrow and spleen play crucial roles in the production, activation, and differentiation of HSCs. Therefore, it is important to explore the effects of aging on HSCs in the bone marrow and spleen. Volcano plots of DEGs in HSCs from the bone marrow and spleen of young and old mice showed that the upregulated DEGs were mostly related to the inflammatory response and the immune system, whereas the downregulated DEGs were mostly associated with cell activation and differentiation processes (Fig. 5A). Further enrichment analysis of these up- and downregulated DEGs revealed that the upregulated genes were mostly enriched in the biological processes of myeloid cell differentiation, inflammatory response, and immune effector process, whereas the downregulated genes were mostly enriched in the biological processes of chromatin organization, cell activation, and mechanisms associated with pluripotency in the HSCs of aging mice (Fig. 5B and S5A). HSCs in the bone marrow of senescent individuals show a tendency toward myeloid differentiation, and we confirmed this conclusion using HSCs from the bone marrow and spleen. By comparing and fitting the myeloid differentiation and lymphoid differentiation scores of cells in young and old mice, respectively, we showed that the cells in aging mice had a more pronounced tendency for myeloid differentiation (Fig. 5C and S5B-C). Moreover, the SASP score of HSCs in old mice was higher than that in young mice and was impacted more by aging than other cells (except for some myeloid cells), indicating an important effect of aging on HSCs (Fig. 5D and 1J).

Since HSCs at different developmental stages are a heterogeneous population [31], we performed cell cycle analysis in order to explore the effects of aging on HSCs in the HIS. Cell cycle analysis showed that these stem cells were distributed in the G1, S, and G2/M phases, compared to young mice where HSCs were predominantly clustered in the G1 phase and had a markedly lower proportion of cells in the S phase, indicating that these HSCs were more in the quiescent phase and less activated with increasing age (Fig. 5E-F). Finally, we sampled bone marrow and spleen cells from 2W and 20M mouse groups to validate the effect of aging on the level of function marker in HSCs using flow cytometry. Compared with CD34- HSCs, CD34 + HSCs are in an activated state and exhibit enhanced differentiation and proliferation capabilities. Experimental bone marrow transplantations in both mice and human cells have confirmed that CD34 + HSCs are able to acquire colony formation ability at a faster rate. Flow cytometry results showed that the proportion of CD34 + HSCs in the bone marrow and spleen of 20M mice was lower than that in 2W mice, suggesting that senescence impairs the differentiation and proliferation capabilities of HSCs in the bone marrow and spleen(Fig. 5G and S5D).

To further investigate the mechanisms underlying HSC changes with aging, we classified HSCs into cluster 0 (high-expression markers: Sell, Dntt, and Ccl9), cluster 1 (high-expression markers: Klf1, Tgfbr3, and Gata1), and cluster 2 (high-expression markers: Hlf, Mllt3, and Mecom) (Fig. 5H and S5E). The results of cell ratio analysis and enrichment analysis of DEGs after aging showed that the number of HSC-0 cells that are mainly enriched in the cellular activation and proliferation pathways which means in the hyperactive state, decreased, but the number of HSC-1 cells that are mainly enriched in the myeloid differentiation and proliferation-related pathways, increased (Fig. 5I-J). And this is consistent with our previous speculations. Enrichment analysis of upregulated and downregulated DEGs revealed that after aging, HSC-0 cells were more involved in the inflammatory response and immune effector processes, whereas HSC-2 cells were less involved in the maintenance of cell pluripotency and differentiation processes (Fig. S5G-H).

Given the high response status observed in the HSC-0 subpopulation during functional analysis, we next focused on validating the surface markers and changes of HSC-0 subgroup. We chose the surface marker CD62L (encoded by Sell) for identify this subset. We confirmed that the proportion of HSC-0 (CD62L + HSCs) was decreased in the bone marrow of old mice compared to young mice using flow cytometry. In contrast, this decrease was not observed in the spleen. These findings suggest age-related changes in HSC populations within specific tissue compartments (Fig. S5I). Moreover, CD150 + CD48- HSCs perform a vital function in preserving the long-term differentiation and proliferation abilities of the HSC population [32]. Our study revealed a decline in the population of CD150 + CD48-CD62L + HSCs in both the spleen and bone marrow with aging (Fig. S5J and S6A). CD62L is crucial for cell migration, homing, and steady-state cell proliferation [33, 34]. In previous studies, it has been observed that bone marrow transplantation in mice utilizing CD62L + HSC sorting results in a faster differentiation into terminal lymphocytes and myeloid cells [35]. Similarly, in post-leukemia bone marrow transplantation patients, sorting CD62 + HSC leads to a quicker colonization of bone marrow and differentiation into neutrophils and platelets [36]. Based on these findings, we hypothesize that the CD62L + HSC subpopulation may consist of highly responsive cells that decrease in number with aging.

CD34 + HSCs and CD201 + HSCs demonstrate a more pronounced multi-lineage repopulation capability in immunocompromised mice [37]. We examined and compared the expression of CD34 and CD201 between CD62L + and CD62L- HSC. The finding indicated that the expression ration of CD34 and CD201 in CD62L + HSCs was higher than that of CD62L- HSCs, and we also observed a decrease in CD62L + CD34 + and CD62L + CD201 + HSCs in aging mice, indicating a decline in the high-response function of CD62L + HSCs with age (Fig. S6B-C). In summary, CD62L + HSCs are a rapidly proliferating and differentiating group of high-functioning populations. However, their high-functioning status decreases with aging. And the highly responsive CD62L + HSCs were obviously downregulated in aging, suggesting that they may play an important role in the aging process. Additionally, CD62L plays a crucial role in determining the differentiation fate of common myeloid progenitor (CMP) cells and granulocyte-monocyte progenitor (GMP) cells [38]. In this study, we utilized flow cytometry to analyze the expression of CD62L in CMP and GMP cells during the aging process. Our findings indicate that aging leads to a reduction in CD62L + CMP and GMP cells, suggesting that aging diminishes the differentiation capacity of these progenitor cells (Fig. S6D).

In conclusion, we classified HSCs in the HIS into three subgroups with different functional states and demonstrated the myeloid differentiation tendency and impaired proliferation and differentiation functions of HSC with aging. And CD62L + HSCs may play an important role in age-related diseases as age-related HSCs.

### Aging alters intercellular interaction patterns

**Fig. 6:**
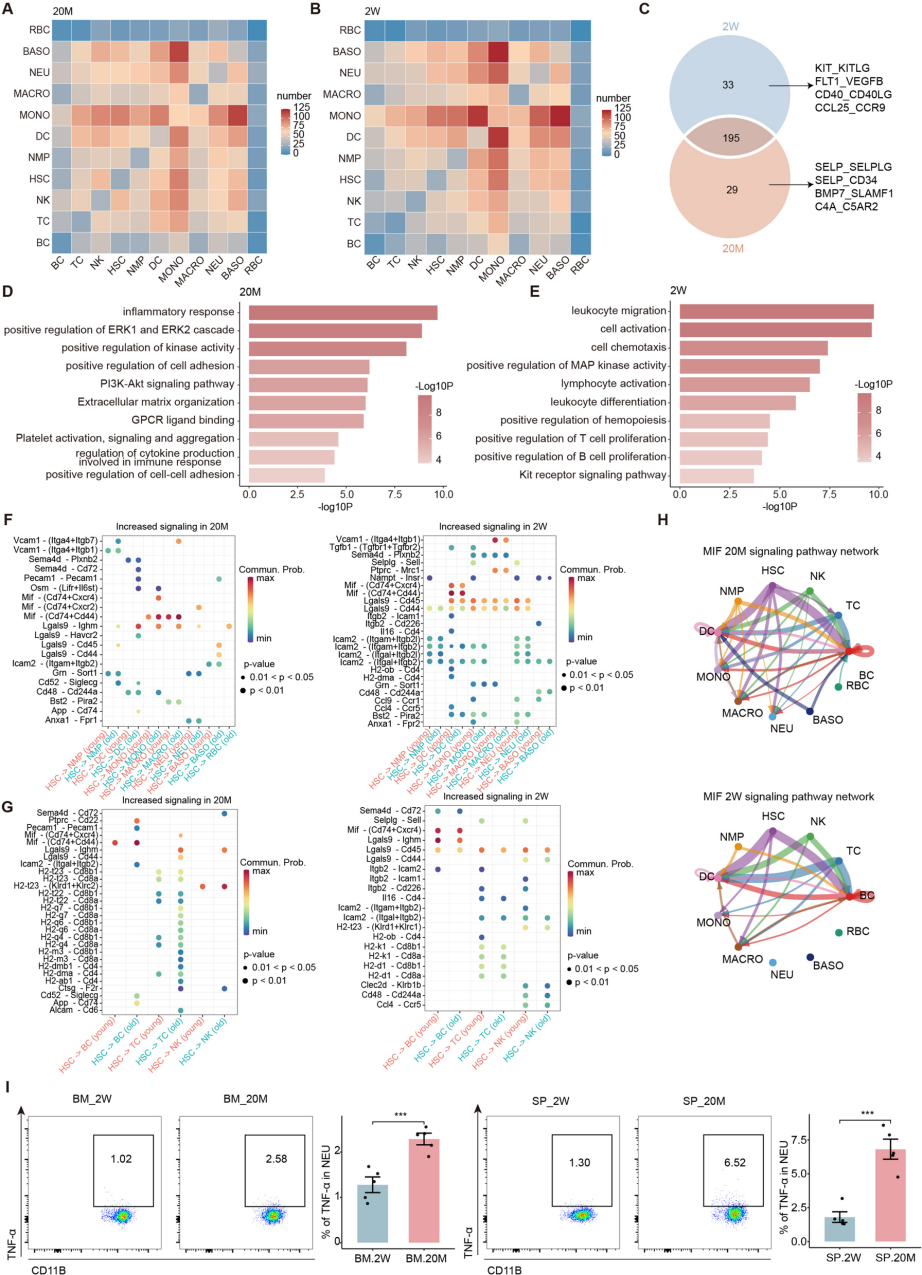
Aging alters intercellular interaction patterns. **A** The heatmap showing the number of communication interaction L-R among immune cell types in 20M. **B** The heatmap showing the number of communication interaction L-R among immune cell types in 2W. **C** Venn diagram showing the interaction of upregulated TFs respectively in two groups. **D** Representative GO biological process and pathways enriched in the upregulated TF-association genes in 20M. **E** Representative GO biological process and pathways enriched in the upregulated TF-association genes in 2W. **F** The dot plot showing the increased L-R signaling of hematopoietic stem cells to myeloid cells in 20M (left) and 2W (right). **G** The dot plot showing the increased L-R signaling of hematopoietic stem cells to lymphocytes in 20M (left) and 2W (right). **H** The circle plot showing the MIF signaling pathway network among immune cell types in 20M (up) and 2W (down). **I** The flow cytometry histograms (left) and column charts (right) showing the level of TNF-α in NEU cells in bone marrow and spleen (*n* = 5/group). Significance in I was calculated using Student’s t test; **P < 0.01, ***P < 0.001, ****P < 0.0001

In the above studies, we found that aging altered the proportion and function of lymphocytes, myeloid cells, and hematopoietic stem cells. However, a simple cell analysis cannot reflect the integral working of the hematopoietic immune system. Therefore, we conducted an intracellular interaction analysis of the hematopoietic immune cells. We analyzed the ligand-receptor (L-R) pairs between hematopoietic immune cells using the CellphoneDB software. Compared to young mice, the interaction between cells in old mice was generally dampened and this was reflected in the decreased communication between myeloid cells, whereas the interaction between DC, T cells, and NK cells heightened (Fig. 6A, B). To explore the specificity of direct cell-to-cell interactions at different ages, we used a Venn diagram to show that L-R pairs were specifically expressed between the two groups. In young mice, the L-R pairs associated with stem cell self-renewal, cell proliferation (KIT_KITLG, FLT1_VEGFB, CCL25_CCR9), and lymphocyte germination (CD40_CD40LG) were highly expressed, whereas in old mice, specific expression of leukocyte adhesion, chemotaxis (SELP_SELPLG), complement secretion (C4A_C5AR2), and inflammatory signals (SELP_CD34) was evident (Fig. 6C). To further explore the different interaction patterns between the young and old groups, we conducted enrichment pathway analysis for genes related to the high expression of L-R pairs in each group. In old mice, pathways related to enhanced immune and inflammatory responses, such as the PI3K-Akt signaling pathway, positive regulation of the ERK1 and ERK2 cascades, and increased secretion of cytokines, were activated (Fig. 6D). Analysis of the expression of inflammatory response-related ligand receptors showed that the interaction between myeloid cells, HSC, and myeloid cells was the most important component in old mice (Fig. S7A). In young mice, the kit receptor signaling pathway was highly enriched, and the pathway related to lymphocyte proliferation and leukocyte differentiation was also highly expressed, and this was also verified by the above findings (Fig. 6E, S7B, and 1H).

CellChat is an important tool for cell-to-cell interaction and pathway visualization. CellChat analysis showed that growth- and development-related pathways (TGFb and BAFF) were upregulated in young mice, while inflammation-related pathways (CD23, CD34, and CD96) were specifically upregulated in old mice (Fig. S7C). To further analyze and verify the relationship between HSCs, lymphocytes, and myeloid cells, we conducted a Cellchat integration analysis in young and old mice. Compared to young mice, the communication probability of L-R associated with inflammation-related MIF was obviously increased in the interaction between HSC and myeloid cells in old mice, while the expression of LGALS9-related L-R pairs was obviously increased in young mice, indicating that the attraction of hematopoietic stem cells to myeloid cells gradually decreases with the progression of aging [39], and this is also consistent with our finding that the proportion of neutrophils in the spleen increased (Fig. 6F, 4H). A similar behavior was observed among HSCs and lymphocytes. In elderly mice, histocompatibility complex-associated ligand receptors were highly expressed in HSC, NK, and T cells, and the expression of macrophage migration inhibitory factor (MIF)-associated L-R pairs increased, whereas in young mice, the L-R pairs related to proliferation and cell aggregation were upregulated (Fig. 6G).

Considering the important role of the MIF pathway, we conducted a visual comparison between young and old mice and found that the interaction between cells increased in old mice and was more significant in myeloid cells, and the genes related to the pathway were also upregulated (Fig. 6H and S7D). At the same time, the TNF signaling pathway [40] that is closely related to MIF, and its passage-related genes were upregulated in elderly mice, especially in the neutrophils (Fig. S7E-F). To confirm our findings, we performed flow cytometry analysis of TNF-α expression in bone marrow and spleen neutrophils and showed that the expression of TNF-α was markedly upregulated in the spleen and bone marrow, being more pronounced in the spleen, consistent with the neutrophilic distribution (Fig. 6I, S7G and 4H). In summary, aging leads to significant changes in interactions among cells of the hematopoietic immune system, as shown by the overall upregulation of immune-inflammatory pathways, especially in myeloid cells, whereas the growth- and development-related pathways are markedly downregulated in aged mice. These findings are important to our overall understanding of the changes in the hematopoietic immune system during aging.

## Discussion

Here, we describe hematopoietic and immune system-specific alterations affected by aging, demonstrating changes in HSCs, immune cell subgroup composition, immune senescence and inflammation, and intracellular interaction-related changes during aging using flow cytometry and scRNA-seq. The main findings are summarized as follows: (1) aging altered the proportion of cells in the hematopoietic and immune systems, increasing the number of terminal state cells and decreasing the number of initial state cells. (2) Aging kept lymphocytes and myeloid cells in a pro-inflammatory and highly differentiated state, resulting in their becoming more involved in terminal immune effector processes; (3) Aging induced reorganization of the hematopoietic and immune systems, with increased differentiation of myeloid and lymphoid immune cells, and this enhanced their immunoinflammatory effects and reduced cellular nativity; (4) With the elevated expression of Hif1α, aging induced an increase in the number and function of neutrophils that resulted in a greater involvement in inflammatory response effects; and (5) Aging induced the reorganization of HSC subsets, especially reduced the CD34 + or CD62L + subsets, resulting in diminished differentiation and proliferation capacity.

In general, the immune system maintains a dynamic balance, characterized by the proper regulation of cell proportion and function [41]. As individuals age, the immune system experiences a decrease in the proportion of lymphocytes and an excessive activation of myeloid cells, resulting in increased immune inflammation. Moreover, as the precursor cells of the entire immune system, HSCs are significantly impacted by aging. This is evident through impaired self-renewal function, an increased tendency towards myeloid differentiation, and decreased proliferation ability [42]. However, local organ or in vitro studies are insufficient to fully analyze the comprehensive changes in both hematopoietic and immune system during aging [43]. Therefore, we have constructed a single-cell landscape of the hematopoietic and immune system related to aging. Our study reveals that aging leads to a decrease in the proportion of lymphocytes, accompanied by a relative increase in terminally differentiated and senescent cells, which upregulates cytokine production (Rora, Ccl5), inflammatory response pathways (S100a8, S100a9), and immune response processes. In contrast, the proportion of myeloid cells not only increases but also the proportion of precursor cells, known as NMP, is relatively elevated. This is accompanied by an increase in SASP and inflammatory response scores (Cxcr2, Hif1a, Cxcl2), with NEU being the main component. Additionally, we observed a reduction in the proportion of HSCs, decreased cell diversity, and a tendency towards myeloid differentiation with aging. These findings indicate that aging disrupts the dynamic balance of the hematopoietic and immune systems. This disruption not only leads to a preference for stem cells to differentiate towards the myeloid system but also results in increased gene expression in myeloid cells involved in immune effector and inflammatory processes, which may be attributed to the deterioration of hematopoietic and immune system functions, as well as the urgent requirement to participate in inflammatory responses during aging [31].

Aging is often accompanied by a decrease in oxygen delivery to all organs and tissues, as well as a decrease in oxygen partial pressure, leading to the development of hypoxia [44]. Aging also alters the metabolic reprogramming of immune cells, leading to altered metabolic pathways such as oxidative phosphorylation and glycolysis, and exacerbating inflammatory responses [45]. However, how aging leads to the metabolic change in HIS cells is still not well defined. In this study, we found aging primarily affects neutrophils, which accumulated in aging mice and were more involved in inflammatory responses and immune effector processes. Neutrophils are activated during inflammatory episodes and downregulated to prevent tissue damage after functioning [46]. Moreover, we demonstrated the role of Hif1α on neutrophils further into the field of aging. We revealed that Hif1α plays a predominant role in neutrophil function during aging by comprehensive analysis of neutrophil transcription factors and DEGs, and this finding was finally validated by flow cytometry. It has been shown that Hif1α induced NF-κB controls neutrophil pro-inflammatory function and survival under hypoxic conditions, leading to persistent inflammation [47, 48]. And elevated levels of Hif1α decrease neutrophil apoptosis and increase their anti-microbial capacity [49]. Hence, we innovatively propose that neutrophils express higher levels of Hif1α following aging in order to better fulfill their role as the first line of host defense against invading pathogens. Previous studies indicate that Hif1α protects the host against bacterial or viral infections by promoting neutrophil survival and improving their phagocytosis. However, the level of Hif1α in neutrophils needs to be well balanced to prevent over-activity that could harm the host. Our study revealed that Hif1α occupies a central position in regulating the effects of aging on neutrophils. Therefore, in the future, precise regulation of Hif1α expression in neutrophils may be a potential target to ameliorate the inflammatory response of aging.

It is well known that HSC aging is a very complex process involving DNA damage, epigenetic drift, ROS-induced oxidative stress, metabolic alterations, and polarity changes [50–54]. A detailed exploration of the effects of aging on HSC will hopefully serve as a powerful tool to slow down or even reverse aging. In this study, we found that aging decreases the proportion of HSCs and decreases related gene expression and pathways. At the same time, aging increased inflammation-related genes and pathways. This suggests that HSC undergo reduced self-renewal, differentiation bias, and inflammatory activation during aging, which is consistent with the findings of Suo et al. [55]. CD34 is a transmembrane phosphoglycoprotein which was considered the important surface marker of HSCs in previous studies. Compared to CD34- HSCs, CD34 + HSCs demonstrate heightened responsiveness and a more rapid differentiation into terminal cells in both in vitro and in vivo studies [56–58]. Based on this, we verified the change in the proportion of CD34 + HSCs after aging by flow cytometry and found that it decreased after aging. Moreover, we clustered HSC into three groups with different functional states. We found that aging decreased the HSC-0 subgroup (with high level of Sell and Dntt) that is mainly responsible for cell activation and proliferation, and increased the HSC-1 subgroup that is involved in myeloid differentiation. In addition, aging interfered with the cell cycle of HSC-0 that maintains highly reactive, causing it to stagnate at the early stages of the cell cycle, further inhibiting its proliferation and differentiation functions. We further validated the decreased proportion of HSC-0 subgroup in bone marrow of old mice based on the expression of CD62L. CD62L encodes a cell surface adhesion molecule which is crucial for cell migration and homing, and the expression of that is also essential for steady-state cell proliferation [33, 34]. This discovery led us to reclassify HSCs, the classically described “the ancestor of the immune system”, into a subpopulation with highly responsive potentiality, which was characterized by CD62L expression. And this is consistent with the study by Cho S et al.[59]. In addition, as mentioned in the previous study, the Mllt3 + HSC-2 subgroup demonstrates a sustained ability for self-renewal and exhibits polymorphism [56–58]. Although the proportion of the HSC-2 subgroup remains relatively stable during aging, both stemness and polymorphism decrease. The study mentioned above suggests that the use of functional markers may be more reasonable compared to traditional surface markers for HSC classification. Currently, genetic modulators (such as TERT, APOE, and FGF21) [60–62], drugs (such as the mTOR inhibitor Rapamycin, Cdc42 inhibitor CASIN, and p38 MAPK inhibitor SB203580) [63–65], and BM niche changes [66] have been used to reverse the decline in HSC function due to aging, and have been shown to be effective. It is reasonable to believe that by more precisely targeting HSC0 and inhibiting HSC1, we will be able to take advantage of the enormous differentiation potential of HSC to ameliorate the degenerative pathologies of multiple tissues and organs caused by aging.

The study also has several limitations. We do not study the influence of gender on the hematopoietic immune system with aging. And we do not study the differences of multi-stage age comparison during the aging process.

## Conclusion

In summary, we combined flow cytometry with single-cell sequencing to establish a comprehensive cellular profile of the hematopoietic and immune systems. We found that aging changes the proportion and function of lymphocytes and myeloid cells, increases the proportion of terminal cells, and involves more immune and inflammatory processes. In addition, we showed that neutrophils are the cell population most affected by aging and identified their target gene, Hif1α. Finally, we demonstrated a myeloid differentiation tendency, impaired stemness, reduced CD34 + or CD62L + subsets, and reduced cellular activity in HSCs after aging. Our findings demonstrate the effects of aging on the HIS and highlight potential targeted therapeutic strategies to delay or even reverse aging.

## Methods

### Mice

Two weeks (2W) and 20 months (20M) old C57BL/6J male mice (2W and 20M mouse groups) were purchased from SPF Biotechnology Co., Ltd. (Beijing, China). Animal experiments were approved by the Institutional Animal Care Committee (Zhongshan Ophthalmic Center, Sun Yat-sen University).

### Cells isolation and preparation

For scRNA-seq, spleen and bone marrow cells from four 2W mice and four 20 M mice were combined into one sample to ensure that a sufficient number of cells were available for sequencing. For the isolation of bone marrow cells, the mice were sacrificed by CO_2_ asphyxiation, the femurs and tibias were dissected, and the muscles and periosteum on their outer surfaces were removed. Bone marrow cells were flushed from the femurs and tibias using cell culture media (RPMI-1640; Gibco) containing 100 IU/mL penicillin, 100 μg/mL streptomycin (Life Technologies), and 10% fetal calf serum (Gemini Bio Products, Sacramento, CA). The cell suspension was filtered through a 40 µm cell strainer (Corning, 431,751) to obtain a single-cell suspension and centrifuged at 1,200 × g for 5 min. For spleen cells, the mice spleens were detached and placed on ice in above RPMI-1640 culture media. The spleen was ground and filtered through a 70 µm cell strainer, and red blood cells were removed with red blood cell lysis buffer (144 mM NH_4_Cl and 17 mM Tris, pH 7.6).

### Flow cytometry analysis

Bone marrow was obtained from the long bones of the limbs of 2W and 20 M mice, and the spleen was removed from the abdominal cavity. These cells were ground and filtered, and erythrocytes were lysed to obtain bone marrow and spleen cells. A Live/dead cell dye (#423,105; BioLegend, San Diego, CA, USA) was used to exclude dead cells. Cells were then stained with the following surface markers antibodies: CD3 PE (#100,245), CD45 APC (#103,112), Cd11b Brilliant Violet 421 (#101,235), Ly6G Brilliant Violet 605 (#127,639), Lineage AF700 (#133,313), SCA-1 APC/CY7 (#108,125), c-kit PE/CY7 (#105,813), CD16/32 PE (#101,307), CD34 BV421 (#152,207), CD62L FITC (#104,405), CD150 PED594 (#115,936), CD48 BV605 (#103,441), CD201 APC (#141,505), CD34 PerCP/Cyanine5.5 (#128,607, BioLegend). For intracellular cytokine staining, the cells were stimulated with 5 ng/mL phorbol myristate acetate, 500 ng/mL ionomycin, and 1 mg/mL brefeldin A (Sigma) at 37 °C and 5% CO_2_ for 5 h, followed by fixation for 45 min and permeabilization for 30 min. Afterwards, cells were stained with antibodies detecting: HIF-1α (#12–7528-82) and tumor necrosis factor (TNF-α) (#12–7321-82, Invitrogen). Finally, the cells were stored at 4 °C overnight in preparation for testing by flow cytometry. After analysis using a flow cytometer (BD LSRFortessa, USA), raw data were analyzed using FlowJo software (version 10.8.1, Tree Star, Ashland, OR, USA).

### scRNA-seq

Single-cell suspensions of bone marrow and spleen cells from 2W and 20 M mice were subjected to the Chromium Single Cell 5’ Library (10 × Genomics, Genomics chromium platform Illumina NovaSeq 6000) Chip Kit, and Gel Bead Kit (10 × Genomics) to generate scRNA libraries following the manufacturer’s protocol. FastQC software was used to check the primary quality of the library and preliminarily process the sequencing data. CellRanger Software (Version 4.0; 10XGenomics), where multiple sequences were separated and barcoded using a counting pipeline in the CellRanger software for the initial processing of sequencing data. The Seurat package (version 4.3.0) was used to perform dimensionality reduction and clustering analysis using default parameters [67]. Quality control was performed to exclude cells with fewer than 200 detected genes and those with a percentage of mitochondrial genes greater than 15%. A total of 16,252 cells (2W, 6,761cells; 20M, 9,491cells) were used for subsequent analysis. ‘NormalizeData’ and ‘ScaleData’ functions were used to logarithmically normalize and scale the data respectively. After ‘Harmony’ was used for batch effect correction [68], we dimensionalized the data by the 'RunPCA' and used 'FindVariableFeatures' to calculate high variability genes. Cell clustering was then performed by 'FindClusters' and 'FindNeighbors.' 'RunUMAP' was used to show cell clustering results in UMAP plots, and 'FindAllMarker' was used to identify preferentially expressed genes in clusters. Table S2 lists the classification markers for all cell subpopulations. And Table S3 lists the cell numbers of all subpopulations in each group.

### Differentially expressed genes (DEGs) analysis

DEGs between the 2W and 20 M groups were identified using the 'FindMarkers' function of the Seurat package (*p*_value_adj < 0.05, |Log2 fold-change|> 0.25. In HSCs, DEGs were identified by *p*-value < 0.05, |Log2 fold-change|> 0.25). Before the DEG analysis, we excluded cell types that were missing or had fewer than three cells in the comparison groups.

### Gene ontology (GO) analysis

GO biological processes and pathway analyses were performed using the Metascape tool to visualize the functional patterns of the DEGs [69]. Of the top 50 enriched GO terms in the different cell subtypes, five to ten GO terms associated with immunity or aging were visualized and analyzed using the ggplot2 R package.

### Pseudotime analysis

Pseudotime analysis was performed using the Monocle2 package [70]. Genes selected for expression in at least 10 cells and DEGs between groups were identified using a *p*-value < 0.05. The two-dimensional trajectory structure was plotted using the 'DDRTree' algorithm and ordered in pseudotime.

### Scoring of biological processes

The R package AUCell (version 1.8.1) [71] was used to score the pathway activity of individual cells. We used an expression matrix to calculate the gene expression ranking in each cell by the "AUCell_buildRankings" function with default parameters. After downloading the canonical path databases from the MsigDB, KEGG, and GO databases, pathways were used to score each cell. The "AUCell_calcAUC" function was used to calculate the area under the curve (AUC) value based on gene expression rankings. Table S1 lists the genes in each gene set.

### Transcription factors regulatory network analysis

A Pyscenic (version 0.12.0) workflow was used to perform transcription factor analysis with default parameters [72]. Transcriptional factors (TFs) of the mm9 database were downloaded from Cistarget (www.resources.aertslab.org/cistarget/) as a reference. The co-expression network of the TFs and target genes was inferred using the GRN function. Using the ctx function in the mm9 database, potential regulons and predicted candidate target genes of TFs were identified by performing a TF motif enrichment analysis. The top 15 upregulated and downregulated TFs were visualized using the R package pheatmap (version 1.0.12).

### Cell cycle evaluation

Forty-three G1/S and 54 G2/M genes that were previously reported as core gene sets were used to conduct a cell cycle analysis with the "CellCycleScoring" function in Seurat [73].

### Cell–cell interactions

Based on scRNA-seq data, we predicted intercellular communications between different cells using the R packet CellChat (version 1.4.0) and CellPhoneDB software (version 3.1.0) with default parameters [74, 75]. Considering that communication is considered nonexistent if ligands and receptors are not detected, we only analyzed the receptors and ligands expressed in at least 10% of specific cells. A heatmap of different cell ligand receptor numbers was visualized using the R package ggplot2, and the expression intensity of ligand receptors between different cells was visualized using the R package Complexheatmap. CellChat was used to analyze and visualize the differences in intracellular ligand receptor intensity between different groups and to analyze and visualize the signal pathway networks.

### Statistical analysis

Statistical analyses and presentations were performed using GraphPad Prism (version 8.0.2; GraphPad Software Inc.) software. Student’s *t* test was used to compare the two groups of data, and nonparametric Spearman’s correlation analysis was used for correlation analysis. When calculating GO biological processes and pathways, *p*-values were obtained by performing hypergeometric tests using the Metascape web tool. Statistical significance was set at *P* < 0.05. ns, not significant, *p < 0.05, **p < 0.01, ***p < 0.001, ****p < 0.0001.

### Supplementary Information

**Additional file 1:** **Fig. S1.** Overall effects of aging on the characterization of Hematopoietic Immune system.

** Additional file 2:** **Fig. S2.** Aging alters the composition and function of T cells in the HIS.

** Additional file 3:** **Fig. S3.** Aging enhances the immune response of B cells, but reduced the response to new antigens.

** Additional file 4:** **Fig. S4.** The number and functional status of myeloid cells, especially neutrophils, obviously increases with aging.

** Additional file 5:** **Fig. S5.** Aging affects the differentiation potential and direction of hematopoietic stem cells in the HIS.

** Additional file 6:** **Fig. S6.** Aging affects the differentiation potential and direction of hematopoietic stem cells in the HIS.

** Additional file 7:** **Fig. S7.** Aging alters intercellular interaction patterns.

** Additional file 8:** **Table S1.** List of pathway-related genes.

** Additional file 9:** **Table S2.** List of the classification markers for all cell subpopulations.

** Additional file 10:** **Table S3.** List of the cell numbers of all subpopulations in each group.

## Data Availability

The scRNA-seq data is deposited in the Genome Sequence Archive in BIG Data Center, Beijing Institute of Genomics (BIG, https://bigd.big.ac.cn/gsa/), Chinese Academy of Sciences. The data of PRG mice was deposited under the GSA Accession No. CRA012362.

## Abbreviations

SASP: Senescence-associated secretory phenotype
NK: Natural killer cells
DCs: Dendritic cells
HSC: Hematopoietic stem cell
HIG: Hematopoietic and immune organs
BC: B cells
TC: T cells
NMP: Neutrophil-myeloid progenitor
MONO: Monocytes
MAC: Macrophages
NEU: Neutrophils
BASO: Basophils
RBC: Red blood cells
UNDEF: Undefined cells
HIS: Hematopoietic immune system
DEGs: Differentially expressed genes
PROTC: Proliferative T cells
CD4NA: Naïve CD4 + T cells
TREG: Regulatory T cells
TH1: T helper 1 cells
TH17: T helper 17 cells
CD8NA: Naïve CD8 + T cells
CD8TEM: CD8 + effector memory T cells
CD8CTL: CD8 + T cells with cytotoxic activity
S100TC: S100 T cells
TEX: Exhausted T cells
PREBC: Precursor B cells
PROBC: Progenitor B cells
NBC: Naïve B cells
S100BC: S100 B cells
PBC: Plasma cells
ABC: Age-associated B cells
L-R: Ligand-receptor
scRNA-seq: Single-cell RNA sequencing
