# Supplementary figures and images for: An aging-related immune landscape in the hematopoietic immune system
Source: Immun Ageing. 2024 Jan 2;21:3. doi: 10.1186/s12979-023-00403-2 (PMC10759628; doi:10.1186/s12979-023-00403-2)

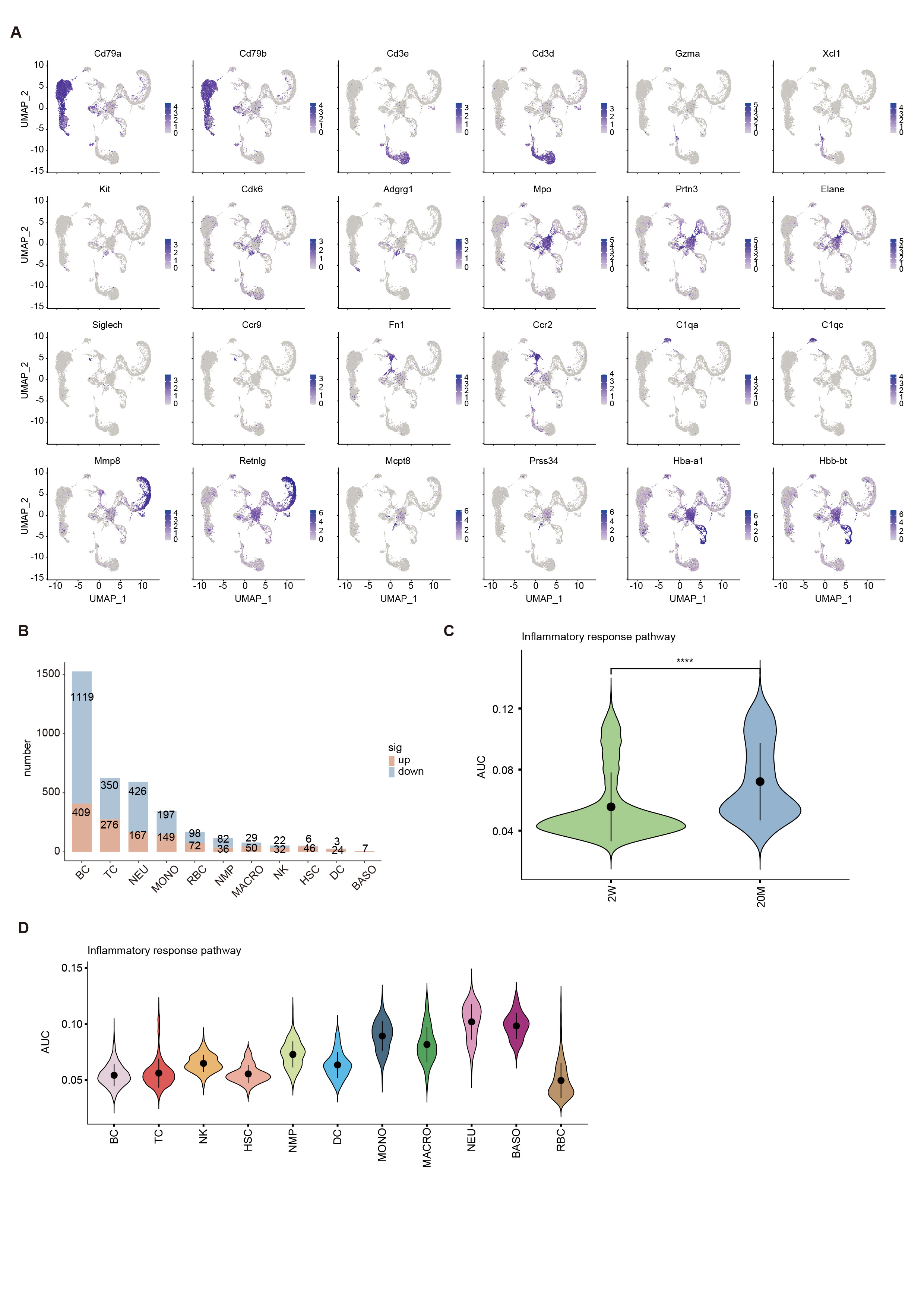

Supplement: Supplementary file 1 — Additional file 1: Fig. S1. Overall effects of aging on the characterization of Hematopoietic Immune system. [file 12979_2023_403_MOESM1_ESM.jpg]

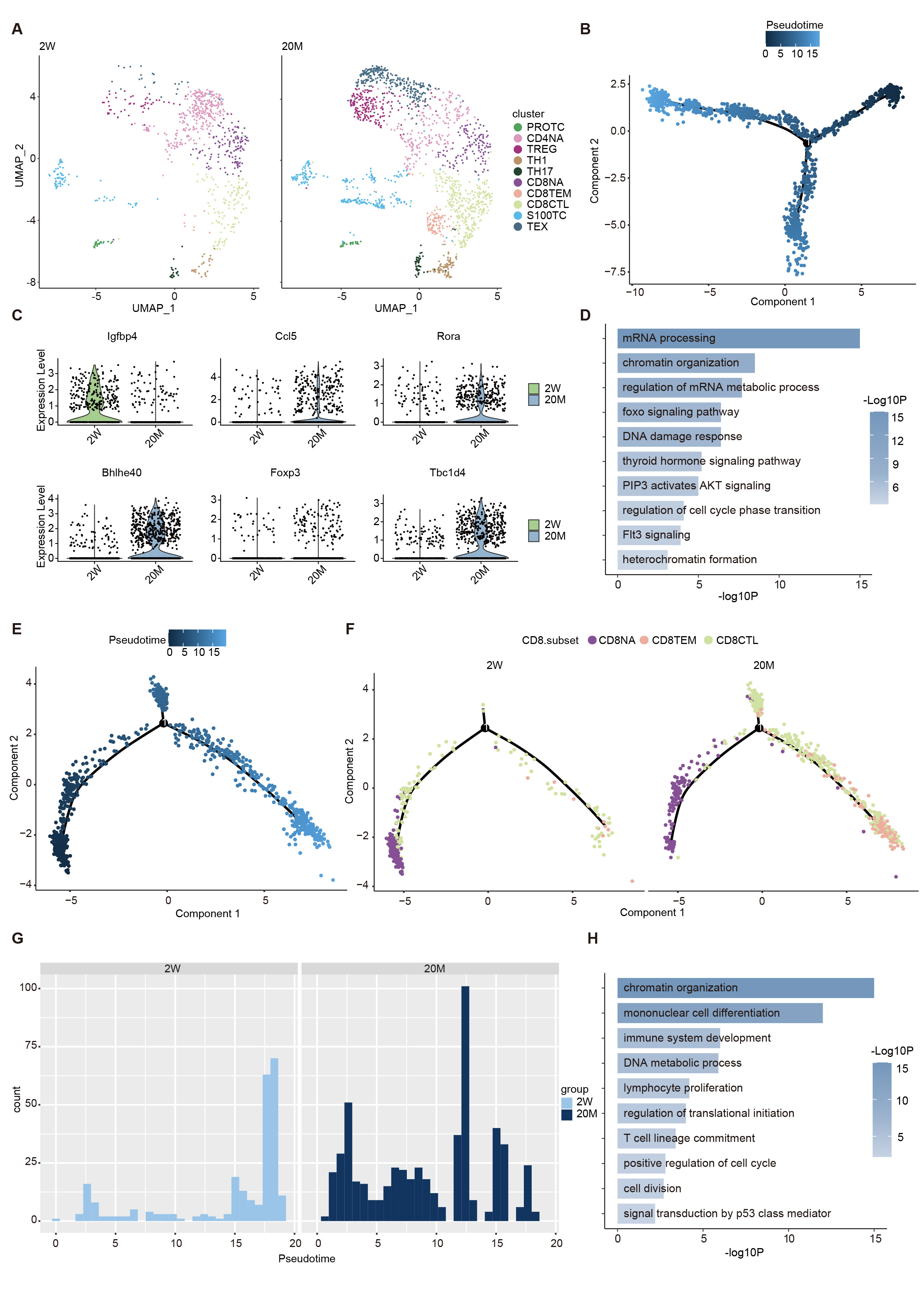

Supplement: Supplementary file 2 — Additional file 2: Fig. S2. Aging alters the composition and function of T cells in the HIS. [file 12979_2023_403_MOESM2_ESM.jpg]

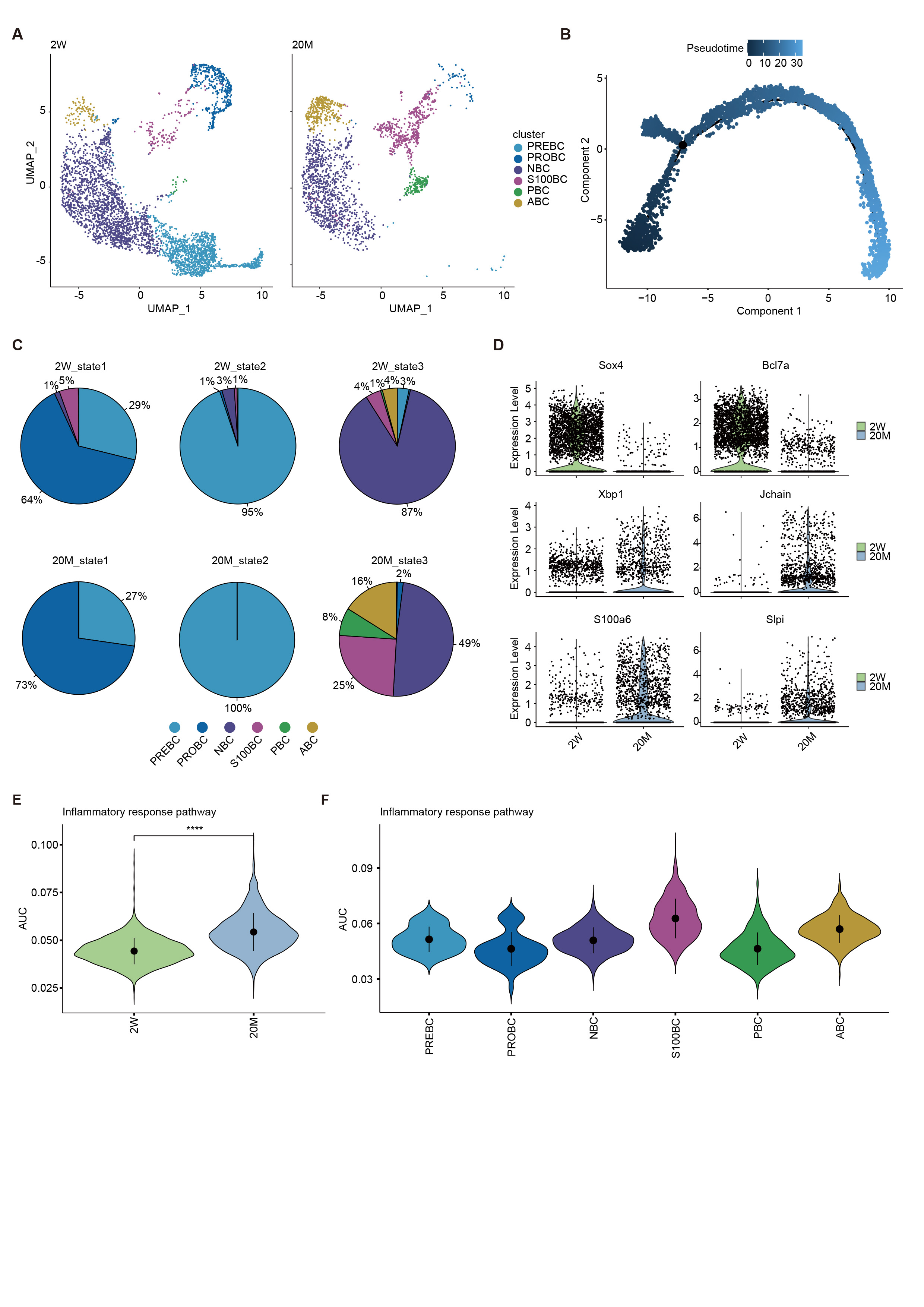

Supplement: Supplementary file 3 — Additional file 3: Fig. S3. Aging enhances the immune response of B cells, but reduced the response to new antigens. [file 12979_2023_403_MOESM3_ESM.jpg]

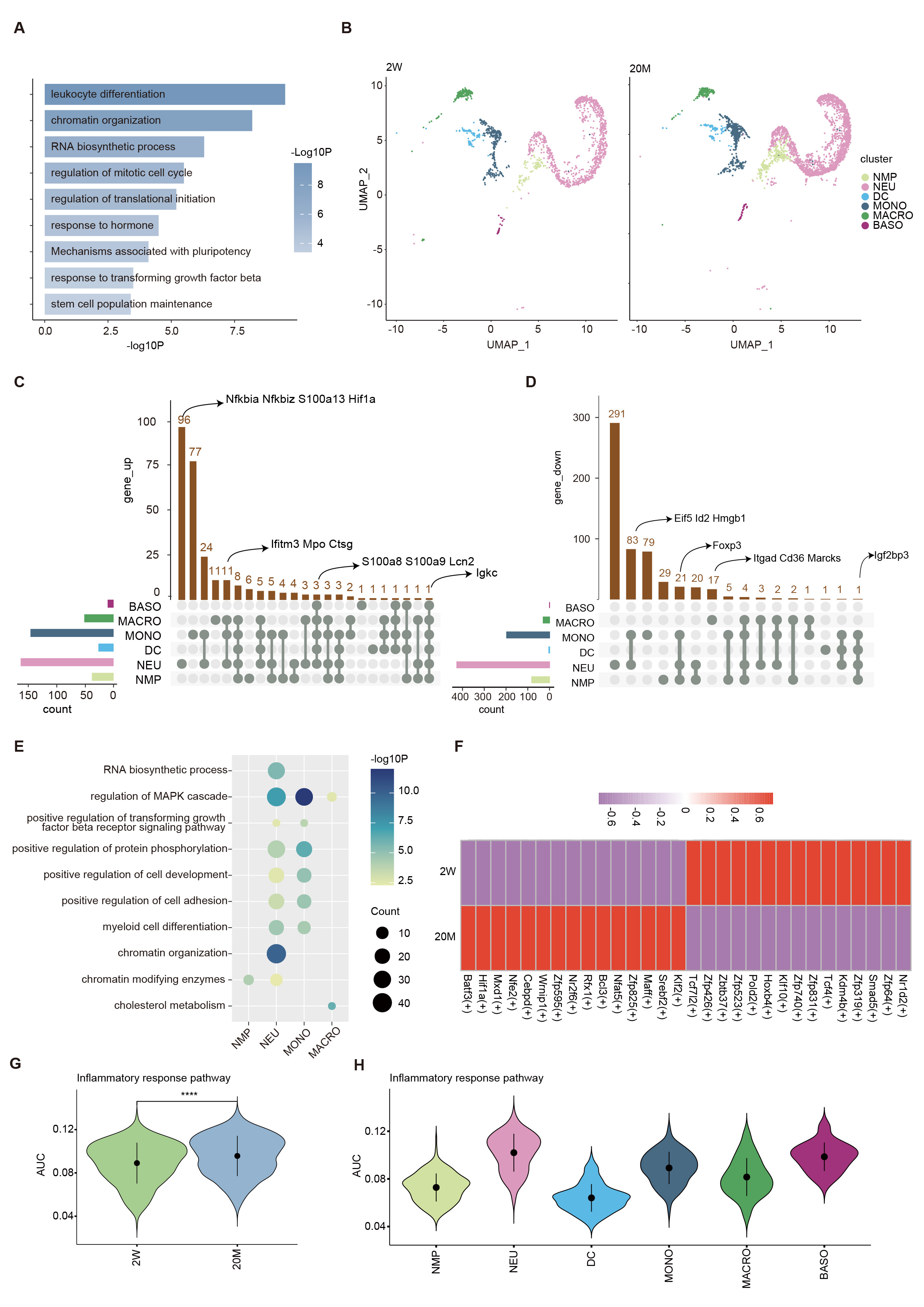

Supplement: Supplementary file 4 — Additional file 4: Fig. S4. The number and functional status of myeloid cells, especially neutrophils, obviously increases with aging. [file 12979_2023_403_MOESM4_ESM.jpg]

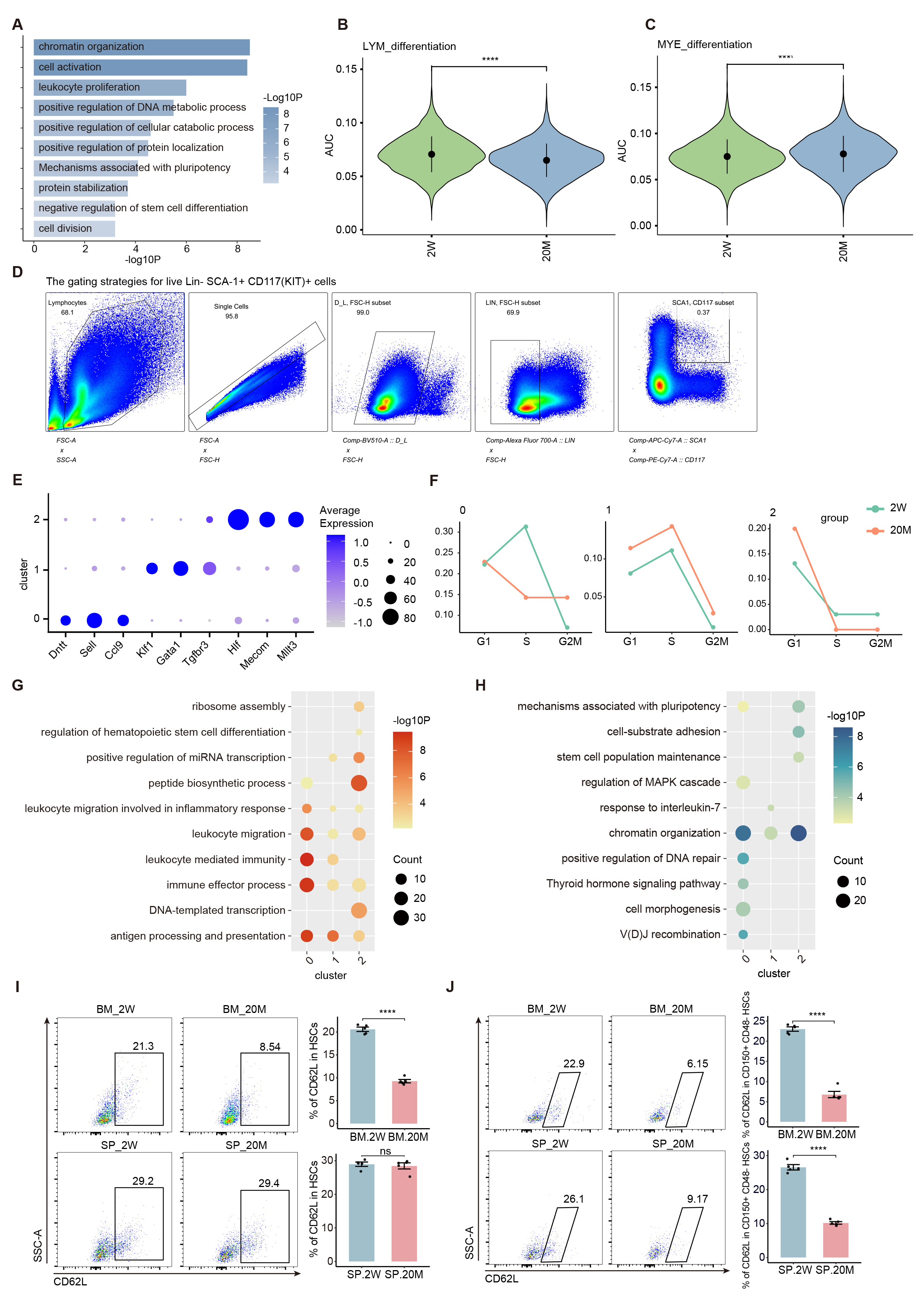

Supplement: Supplementary file 5 — Additional file 5: Fig. S5. Aging affects the differentiation potential and direction of hematopoietic stem cells in the HIS. [file 12979_2023_403_MOESM5_ESM.jpg]

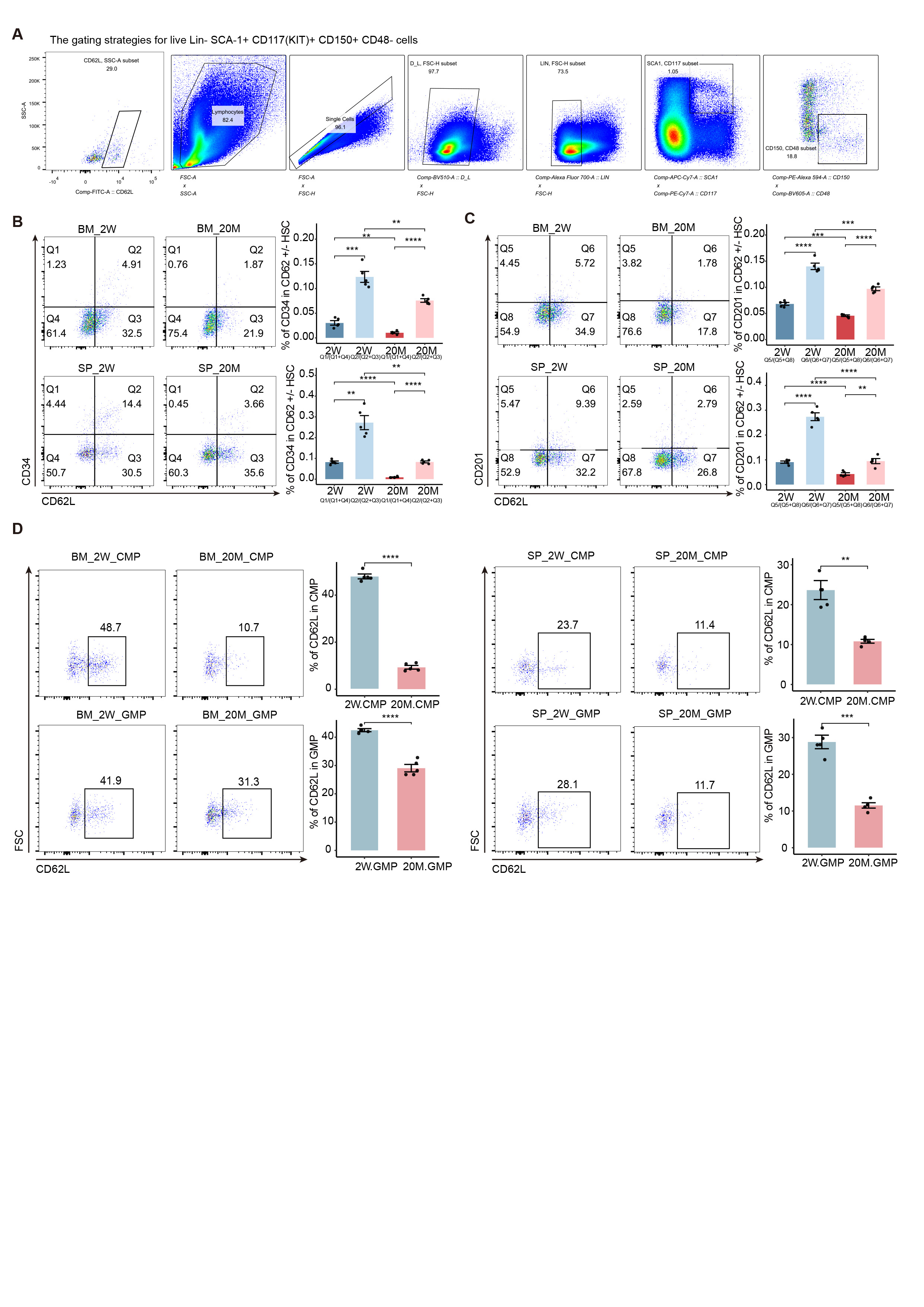

Supplement: Supplementary file 6 — Additional file 6: Fig. S6. Aging affects the differentiation potential and direction of hematopoietic stem cells in the HIS. [file 12979_2023_403_MOESM6_ESM.jpg]

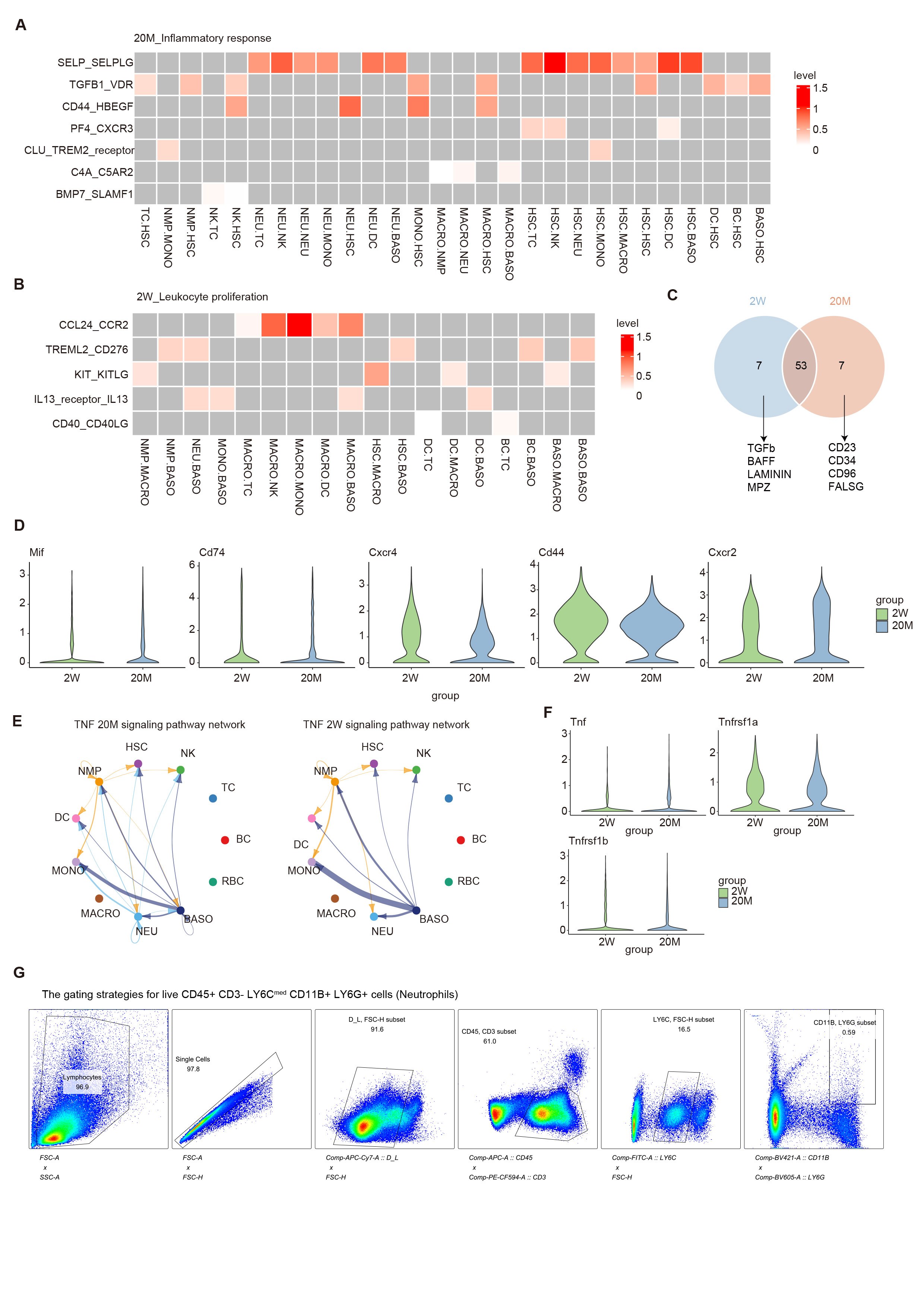

Supplement: Supplementary file 7 — Additional file 7: Fig. S7. Aging alters intercellular interaction patterns. [file 12979_2023_403_MOESM7_ESM.jpg]
